# Finger Tracking Reveals the Covert Stages of Mental Arithmetic

**DOI:** 10.1162/OPMI_a_00003

**Published:** 2017-02-01

**Authors:** Pedro Pinheiro-Chagas, Dror Dotan, Manuela Piazza, Stanislas Dehaene

**Affiliations:** 1 Cognitive Neuroimaging Unit, CEA DRF/I2BM, INSERM, Université Paris-Sud, Université Paris-Saclay, NeuroSpin center, France; 2 École Doctorale Cerveau-Cognition-Comportement, Université Pierre et Marie Curie, France; 3 Collège de France; 4 Language and Brain Lab, School of Education and the Sagol School of Neuroscience, Tel Aviv University; 5 Center for Mind/Brain Sciences University of Trento

**Keywords:** arithmetic, finger tracking, serial processing, problem-size effect, operational momentum effect

## Abstract

We introduce a novel method capable of dissecting the succession of processing stages underlying mental arithmetic, thus revealing how two numbers are transformed into a third. We asked adults to point to the result of single-digit additions and subtractions on a number line, while their finger trajectory was constantly monitored. We found that the two operands are processed serially: the finger first points toward the larger operand, then slowly veers toward the correct result. This slow deviation unfolds proportionally to the size of the smaller operand, in both additions and subtractions. We also observed a transient operator effect: a plus sign attracted the finger to the right and a minus sign to the left and a transient activation of the absolute value of the subtrahend. These findings support a model whereby addition and subtraction are computed by a stepwise displacement on the mental number line, starting with the larger number and incrementally adding or subtracting the smaller number.

## INTRODUCTION

Despite decades of research in cognitive arithmetic, how the brain performs elementary arithmetic calculations remains largely unknown. The widely replicable problem-size effect is the finding that response times (RTs) and error rates increase as a function of the size of the operands to be added or subtracted. By investigating the properties of the problem-size effect across operations and during development, researchers have proposed different cognitive models of arithmetic (Zbrodoff & Logan, 2005).

In their seminal study, Groen and Parkman (1972) found that the best predictor of single-digit addition RTs in first graders was the size of the smaller operand (*min*). They proposed that children use a counting strategy to solve additions by starting from the larger operand and then incrementing it with the *min*, with a slope of about 410 ms per unit. A much smaller slope, however, was found in adults (20 ms/unit). This seemed too fast for a counting strategy, and the authors proposed that adults directly retrieve the results from long-term memory.

Fact retrieval is thought to be a dominant strategy in adults, but some data show that it is also supplemented by other strategies (Lefevre & Kulak, 1994; Siegler, 1987; Zbrodoff & Logan, 2005). For instance, Butterworth, Zorzi, Girelli, and Jonckheere (2001) proposed that, given the commutativity of addition, only half of the table may be stored in long-term memory (problems in which the first operand is larger than the second, which could be progressively committed to memory as a result using the *min* counting strategy at a younger age). To solve problems presented in the opposite order (e.g., 2 + 7), participants would reorder the operands prior to retrieval. Adults’ RTs for additions were indeed found to be higher when the first operand was smaller, presumably due to this additional reordering stage.

Some models propose that arithmetic problems are solved by quantity manipulation, possibly relying on a “mental number line” (Dehaene, Spelke, Pinel, Stanescu, & Tsivkin, 1999). Considerable research indicates that children and adults possess such a space-like left-to-right numerical representation, and that arithmetic may involve internal movements on this representation (Barrouillet & Thevenot, 2013, Dehaene & Changeux, 1993; Fayol & Thevenot, 2012; Knops, Thirion, Hubbard, Michel, & Dehaene, 2009; Knops, Viarouge, & Dehaene, 2009; Mathieu, Gourjon, Couderc, Thevenot, & Prado, 2016; McCrink, Dehaene, & Dehaene-Lambertz, 2007; Restle, 1970, Uittenhove, Thevenot, & Barrouillet, 2016). Accordingly, mental arithmetic causes spatial biases similar to the SNARC (spatial-numerical association of response codes) effect with single numbers (Dehaene, Bossini, & Giraux, 1993): addition draws attention and eye movements toward the right side of space, and subtraction toward the left (Knops, Viarouge, et al., 2009; Mathieu et al., 2016; Pinhas, Shaki, & Fischer, 2014). There is also a tendency to overestimate the results of addition and to underestimate the results of subtraction, which can be interpreted as an excessive motion on the number line and has therefore been termed the “operational momentum” (OM) effect (Knops, Viarouge, et al., 2009; McCrink et al., 2007). However, it is still unknown whether those effects betray a genuine use of the number line during calculation, or are merely an automatic attraction to the result after it has been calculated.

Progress in understanding mental arithmetic is impeded by the fact that RTs and error rates provide only a single summary measure of the entire calculation process, blind to the succession of intermediate stages. Here, we introduce an online measurement method that addresses this temporal dissection problem: continuous finger tracking (Dotan & Dehaene, 2013, 2016; Song & Nakayama, 2009; see also Freeman & Ambady, 2010, for a similar approach with mouse tracking). Participants solved single-digit additions and subtractions on a tablet computer, and responded by pointing to the position of the result on a horizontal number line ranging from 0 to 10, while their finger trajectory was continuously monitored. By identifying which cognitive factors affect finger location at each time point, we aimed to answer several questions: Are the two operands processed serially or in parallel? Is there a stage whose duration increases linearly with the size of the numerical quantities, as implied by models of counting (*min*) or motion on the number line? Can we visualize a reordering of the two operands when solving additions, as predicted by the comparison model? And can we determine the moment when the visuo-spatial biases underlying addition and subtraction occur?

## METHOD

**Figure 1.**
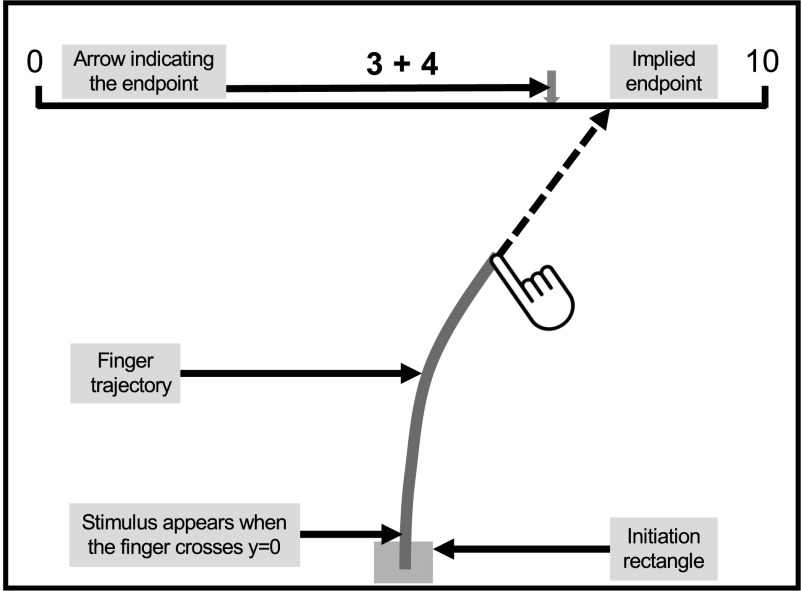
Task and screen layout.

Thirty right-handed French adults aged between 20 and 45 (*M* = 24, *SD* = 5) participated in the study. The participants saw a series of single-digit addition and subtraction problems on a tablet computer and were instructed to point at the position of the result on a horizontal number line marked with 0 and 10 at its extremities (see Figure 1). On each trial, participants first touched an initiation rectangle, which made a fixation cross appear above the middle of the number line. When participants started moving their finger toward the number line, an arithmetic operation appeared at fixation for 250 ms. Participants then continued moving their finger to what they judged to be the position of the result. When the finger reached the number line, a feedback arrow indicated the location where the finger landed.

Using an Apple iPad 2 in landscape orientation, finger position was sampled at 60 Hz (1 ms accuracy), resampled at exactly 100 Hz using cubic spline interpolation, and smoothed (Gaussian, *σ* = 20 ms). For each time point, we calculated the instantaneous direction as the vector difference between the finger coordinates at times *t*−10 ms and *t*, and the *implied endpoint* (*iEP*) as the position on the number line that the finger would reach if it kept moving straight in this direction.

The experiment included two blocks, presented in random order, both mixing single-digit additions and subtractions (individual digits were also presented, but are not reported here). The blocks were designed to control for possible confounds in the analyses of the OM effect. Block 1 included all single-digit addition and subtraction problems with matched operands between 1 and 9 (e.g., 4 + 3 and 4 − 3), resulting in 25 additions with larger-first operands (denoted *L* + *S*, where *L* is the larger and *S* the smaller number) and 25 subtractions (*L* − *S*). Each problem was repeated six times, for a total of 300 trials. In this block, addition results are generally larger than subtraction results, and, thus, the presence of an OM effect could be due to this bias. As a control, we therefore used matched results in block 2. We started from the 54 additions and 45 subtractions with operands ranging from 0 to 9 and results ranging from 1 to 9 (thus including *L* + *S*, *S* + *L*, and *L* − *S* problems). If each problem appeared exactly once, the distribution of addition and subtraction results would again be asymmetrical. Therefore, we overrepeated some problems to obtain exactly 20 addition and 20 subtraction trials for each of the results 1–9 (total of 360 trials). By construction, block 2 contained all the problems presented in block 1. Therefore, the OM effect could be investigated in a most unbiased manner by restricting the analysis to addition and subtraction problems from block 1 (with identical operands) that were presented in block 2 (with equalized distributions of response locations). The two blocks also allowed us to test the stability of our findings.

Trajectory analysis followed the method introduced in Dotan and Dehaene (2013). First, for each participant, one regression was run per time point in 30-ms intervals. The dependent variable was the *iEP*. Predictors were the two operands, the operator (− or +, coded as −1 and 1), the spatial-reference-points-based bias function (SRP; see Supplementary Materials), and the result of the previous trial. The latter two predictors were added in all regressions in order to capture maximal variance, as they were significant in previous studies (Dotan & Dehaene 2013). As their effect was virtually identical in all conditions, they are only reported in Supplemental Materials (Pinheiro-Chagas, Dotan, Piazza, & Dehaene, 2017).

At a second stage, we compared the *b* values of the different predictors (paired *t* test or repeated-measures ANOVA). To examine whether a given predictor has a significant group-level effect in each time point, we compared the participant’s *b* values to zero using one-sample *t* test. Each *b* value (also called regression weight) provides a quantitative measure of the extent to which each element of the operation influences the finger trajectory at each time point. The reported *p* values are one-tailed, since we assumed that all effects of all predictors included would be positive.

## RESULTS

### Movement Time

**Figure 2.**
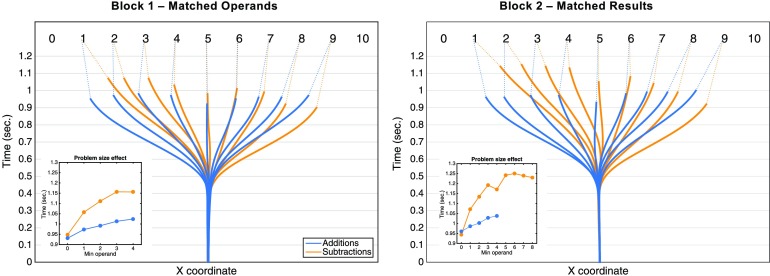
Reconstructed trajectories per result and per operation, averaged across subjects. Insets show how movement time increases as a function of the *Min operand*.

We first analyzed the overall movement time (MT) from stimulus presentation to number-line touch (equivalent to RT in oral calculation tasks). In both blocks, MT was longer for subtractions compared to additions *L* + *S* (block 1: additions *L* + *S*: *M* = 966 ms, *SD* = 118 ms; subtractions: *M* = 1040 ms, *SD* = 138 ms; *t*_(29)_ = −14.72; *p* < .001; *d* = −.58. Block 2: additions: mean = 982 ms, *SD* = 127 ms; subtractions: *M* = 1072 ms, *SD* = 156 ms; *t*_(29)_ = −12.55; *p* < .001; *d* = −.64). In agreement with the COMP model of Butterworth et al. (2001), additions *L* + *S* were solved 14 ms faster than additions *S* + *L* (block 2: additions *L* + *S*: *M* = 976 ms, *SD* = 128 ms; additions *S* + *L*: mean = 990 ms, *SD* = 125 ms; *t*_(29)_ = −5.23; *p* < .001; *d* = −.11). To investigate the problem-size effect, we performed a stepwise multiple regression with *MT* as the dependent variable and the *Min operand*, *Max operand*, and *Result* as predictors, separately for additions and subtractions in each experimental block. The best predictor of MT was always the *Min operand*(insets in Figure 2). For additions, the *Min operand* had a *b* value of 26 ms per unit (*p* < .001) in block 1 and of 21 ms per unit (*p* < .001) in block 2. The *Max operand* had a small but significant negative effect in block 1 (*b* = 8 ms; *p* <.001), but a null effect in block 2 (*p* = .895). Finally, the *Result* had a null effect in both blocks (*p* > .8). For subtractions, the *Min operand* had a *b* value of 62 ms per unit (*p* < .001) in block 1 and of 44 ms per unit (*p* < .001) in block 2. The *Max operand* had a null effect (*p* > 1) in both blocks. Finally, the *Result* had a small but significant effect in both blocks (*b* = −4.53 ms; *p* = .002 in block 1 and *b* = −7.07 ms; *p* < .001 in block 2). Overall, the dominant effect of the *Min operand* is therefore consistent across both blocks and operations.

### Accuracy

Next we analyzed response accuracy, that is, the location where participants landed their finger (endpoint) in relation to the ideal location. The *endpoint error* is the absolute difference between the endpoint and the correct result (in numerical units). The *endpoint bias* is the difference between the endpoint and the correct result, with positive values indicating rightward bias. Subtractions produced larger endpoint errors, but this difference only reached statistical significance in block 1 (block 1: additions L+S: *M* = .43, *SD* = .13; subtractions: *M* = .47, *SD* = .180; *t*_(29)_ = −2.47; *p* = .019; *d* = −.26; block 2: additions L+S: *M* = .47, *SD* = .150; subtractions: *M* = .48, *SD* = .180; *t*_(29)_ = −0.20; *p* = .841, *d* = −.06). Additions L+S produced a slight greater leftward endpoint bias compared to subtractions, but this difference was only significant in block 2 (block 1: additions *L* + *S*: *M* = −.12, *SD* = .16; subtractions: *M* = −.06, *SD* = .170; *t*_(29)_ = −1.24; *p* = .226; *d* = −.37; block 2: additions L+S: *M* = −.22, *SD* = .14; subtractions: *M* = .02, *SD* = .160; *t*_(29)_ = −6.92; *p* < .001, *d* = −1.63). Note that this effect is the opposite of the OM effect. A comprehensive analysis of the full time course of the OM effect is presented further below (see Figure 5), but here we simply note that in block 2 subtractions had overall larger first operands as compared to additions (in order to yield matched results), which may have dragged responses further to the right for subtractions. No significant differences in endpoint error or endpoint bias were found between additions *L* + *S* and additions *S* + *L* (block 2, endpoint error: additions *L* + *S*: *M* = .45, *SD* = .15; additions S+L: *M* = .47, *SD* = .160; *t*_(29)_ = −1.95, *p* < .061; *d* = −.13; endpoint bias: additions L+S: *M* = −.19, *SD* = .13; additions S+L: *M* = −.20, *SD* = .16; *t*_(29)_ = 0.59; *p* = .553; *d* = .07). Finally, with respect to the problem-size effect, the *Min* operand, which was the best predictor of movement times, was also a significant predictor of endpoint error in both additions and subtractions in both blocks (block 1: additions: *b* = .41, *p* < .001, subtractions: *b* = .43, *p* < .001; block 2: additions: *b* = .44, *p* < .001, subtractions: *b* = .42, *p* < .001).

### Trajectory Dynamics

**Figure 3.**
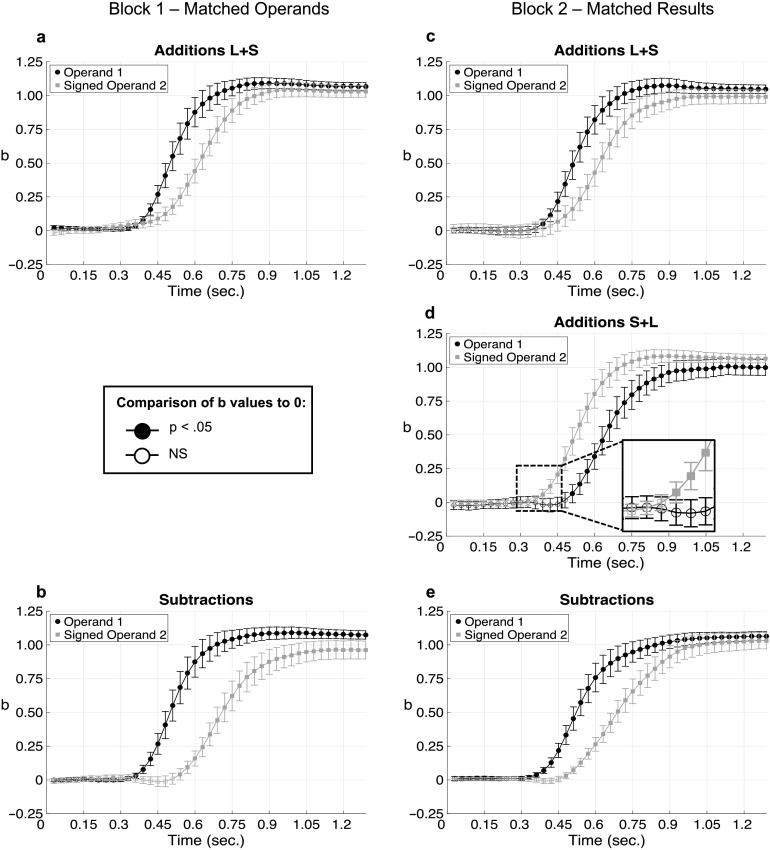
Time course of the regression effects in block 1 (a, b) and in block 2 (c, d, e) per condition. The *b* values were averaged over participants and plotted as a function of time. The *b* values were compared to zero (*t* test), black dots denote *p* < .05. Error bars represent 95% confidence intervals.

Next, we analyzed the full trajectories (Figure 3). The first question we considered was whether the operands are processed in parallel or serially. In additions with larger operand first (*L* + *S*), regressions indicated that the finger first moved according to the first operand, and only then a significant effect of the second operand emerged. The first operand has a significantly higher effect compared to the second starting at 420 ms (*p* = .017) in block 1 and at 450 ms in block 2 (*p* < .001) and this difference remained significant until the end of the trajectory.

Remarkably, for additions with smaller operand first (*S* + *L*), the order was reversed: the second operand (larger number) had a higher weight compared to the first operand (smaller number) from 390 ms on (*p* < .047), compatible with the assumption that the two operands are reordered prior to effecting the finger movement. This pattern remained stable until the end of the trajectory. In fact, the second operand (larger) deviated from zero at 390 ms (*b*[signed_op2] > .057, *R*^2^ > 0.036, *t*_(29)_ > 2.998, *p* < .034), 120 ms before the effect of the first operand at 510 ms (*b*[op1] > .064, *R*^2^ > .020, *t*_(29)_ > 1.943, *p* < .030).

In subtractions, a serial effect was again observed. The first operand had a higher effect than the second during almost the entire trajectory (from 390 ms on in block 1 and from 360 ms on in block 2). Both operations therefore indicate that the operands are processed serially: participants start processing the larger operand followed by the smaller, regardless of the order in which they appeared. Additional analyses (see Supplemental Materials, Pinheiro-Chagas et al., 2017) revealed that the finger first moved according to the larger operand *L* at the same time in all arithmetic operations, and then a correction was introduced for the smaller operand *S* at different delays (*L* + *S* < *S* + *L* < *L* − *S*).

**Figure 4.**
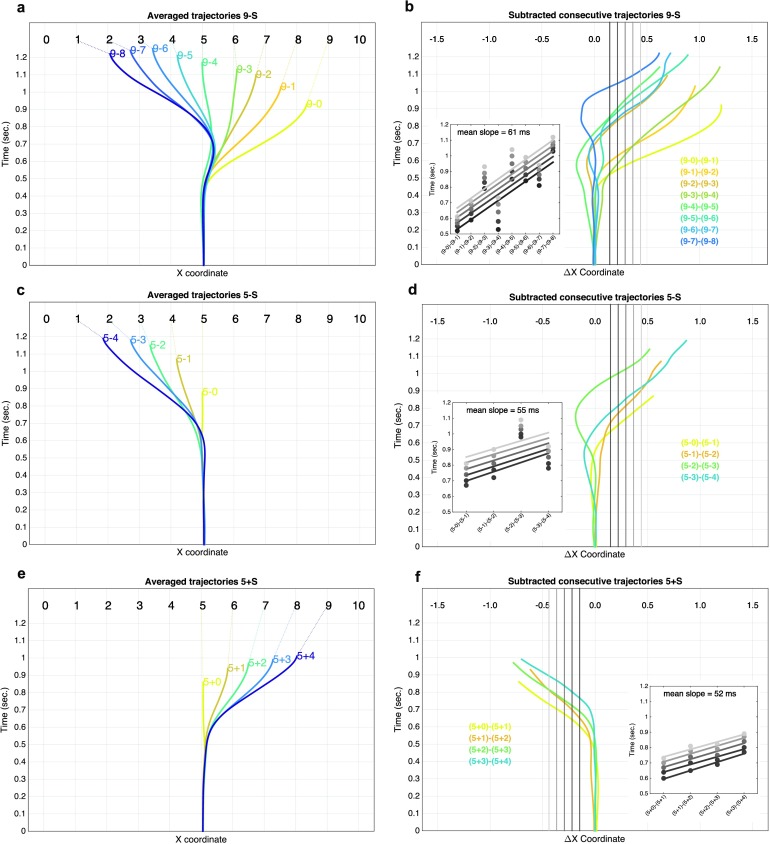
Reconstructed trajectories per operation averaged across subjects (a, c, e) and subtracted consecutive trajectories (b, d, f). Insets show the divergence time as a function of the number being subtracted, for different Δ*X coordinate* threshold.

To directly visualize this serial processing pattern, we returned to the individual trajectories for specific problems. Figure 4a shows the example of subtraction problems “9 - *S*” (where *S* ranges from 0 to 8), in which we could investigate the full spectrum of results 1–9. The plot shows that the finger first deviates toward the right (i.e., in the direction of the larger operand 9) and then to the correct result. Additionally, the latter correction seems to be progressive, as if the finger goes through intermediate stages. This is most clearly seen for the problem 9 - 8: the trajectory first coincides with that for 9 - 0, then 9 - 1, 9 - 2, and so on.

To characterize these effects, we subtracted consecutive trajectories (9 - 7) - (9 - 8), (9 - 6) - (9 - 7), and so on, and plotted the resulting difference trajectories, thus revealing the time course of their divergence (Figure 4b). We then examined whether the divergence times increased with the *Min Operand.*Divergence time was measured as the moment when the difference in finger location (Δ*x*) achieved a threshold value. Regardless of the particular choice of thresh- old, regressions indicated that the divergence time increased with the number being subtracted (*p* < .001) with a stable slope of about 60 ms per additional unit. Similar results were found for subtractions 8-*S*, 7-*S*, 6-*S*, and so on (slopes = 75, 66, 68 ms, respectively). In order to test whether the same pattern was also present in additions, we selected the problems 5 + *S* and 5 − *S*, since they have the larger range of *S* that can be matched between operations. Both subtractions 5 − *S* (mean slope = 55 ms) and additions 5 + *S* (mean slopes = 52 ms) showed a progressive deviation from the larger operand, proportional to the size of the *Min Operand*.

**Figure 5.**
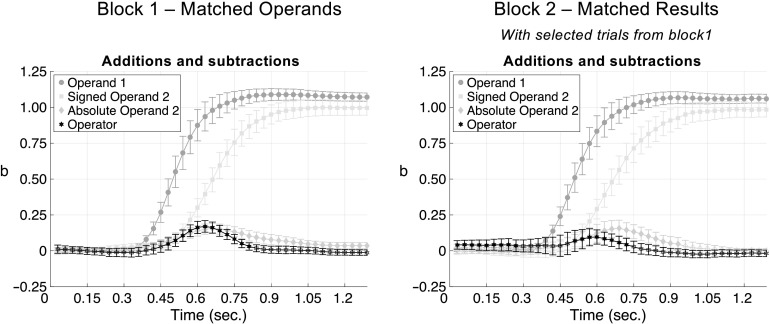
Time course of the regression effects in block 1 (a) and in block 2 with the selected trials of block 1 (b) for additions and subtractions together.

To investigate the OM effect, we pooled additions and subtractions (Figure 5). In block 1, the operator had a significant transient effect from 480 ms to 810 ms (*b*[operator] > .048, *R*^2^ > .005, *t*_(29)_ > 2.335, *p* < .013), reaching its peak value at 630 ms (*b*[operator] = .170, *R*^2^ = .014, *t*_(29)_ = 8.673, *p* < .001). The effect was positive, indicating that an addition sign transiently distorts the trajectory toward the right side of the number line, and a subtraction sign toward the left side, as expected from the OM effect. The results remained essentially unchanged when we analyzed the same arithmetic problems as in block 1, but now using the data from block 2, that is, with an unbiased distribution of result size for addition and subtraction. There was a significant positive transient effect of the operator (210 ms) from 510 ms to 720 ms. (*b*[operator] > .069, *R*^2^ > .009, *t*_(29)_ > 1.913, *p* = .033), reaching its peak *b* value at 600 ms (*b*[operator] = .096, *R*^2^ = .008, *t*_(29)_ = 3.973, *p* < .001) and then progressively dropping until losing statistical significance at 750 ms.

An interesting visualization of the OM effect is provided by addition and subtraction problems involving zero (*x* + 0 and *x* − 0 problems), which can be directly compared with single-digit controls where *X* alone is presented. As shown in Figure S3 (Pinheiro-Chagas et al., 2017), when plotting the implied endpoints per time, subtractions show a systematic transient leftward bias, additions a rightward bias, and single digits tend to fall in between. This variant of the OM effect was statistically confirmed in a regression analysis comparing additions *x* + 0 and subtractions *x* − 0 (insets in Figure S3 (Pinheiro-Chagas et al., 2017)).

Interestingly, at about the same time as the effect of the operator, we observed a significant transient effect of the absolute value of the second operand in both blocks (block 1: from 330 ms on *b*[abs_2op] = .017, *R*^2^ = .003, *t*_(29)_ = 1.994, *p* < .028, peak *b* at 510 ms; block 2 from 510 ms to 960 ms, *b*[abs_2op] > .078, *R*^2^ > .012, *t*_(29)_ > 3.233, *p* < .001; peak *b* at 690 ms). The effects of *b*[op1] and *b*[signed_op2] were highly similar to the ones found in previous regression analyses, with the effect of *b*[signed_op2] delayed as compared to *b*[op1].

In brief, the temporal dynamics of the OM effect revealed a spatial bias induced by the operator coinciding with the time that participants are processing the second operand. Furthermore, participants also seemed to transiently represent the absolute value of the subtrahend.

## DISCUSSION

By continuously measuring finger position in a novel calculate-and-point task, we obtained a detailed picture of the processing stages underlying addition and subtraction. Importantly, overall movement time and accuracy replicated previous findings in cognitive arithmetic, indicating that our task did not depart radically from previous oral calculation tasks. Subtractions were solved slower and less accurately than additions, and within additions, those with a smaller first operand were solved 14 ms slower than those with a larger first operand (Butterworth et al., 2001, reported a 13 ms difference). This effect is not trivial, since it runs opposite to what could have been predicted in the light of spatial-numerical congruency effects, such as the SNARC (Dehaene, Bossini, & Giraux, 1993)—that is, that additions *S* + *L*, where digits are in the proper left-to-right order, would be solved faster than *L* + *S*.

In line with Barrouillet and Thevenot (2013) and Uittenhove et al. (2016), we also detected a robust problem-size effect in single-digit additions, even with operands in the range 0–4. The best predictor of movement time was the smaller operand (*Min*) in both additions and subtractions. The slopes that we found for additions (21 ms per unit in block 1 and 26 ms in block 2) match the slope of 26 ms reported by Barrouillet and Thevenot (2013). Finally, subtractions showed a higher problem size effect than additions, also consistent with previous studies (Seyler, Kirk, & Ashcraft, 2003).

The main contribution of our study is to investigate the covert processing stages underlying arithmetic calculations. Regression analyses on instantaneous finger direction allowed us to uncover two new effects. First, in both additions and subtractions, the operands were processed in a serial way: participants started processing the larger operand, then the smaller operand, regardless of the order in which they were presented. In particular, we could directly observe a reordering in S + L additions, as predicted by the COMP model (Butterworth et al., 2001). The presence of an additional reordering stage may also explain why *S* + *L* additions were slower than *L* + *S* additions.

Comparing each predictor across additions and subtractions further revealed that the larger operand is processed at about the same time in all conditions, and then a correction is introduced for the smaller operand, at a variable delay (additions *L* + *S* < additions *S* + *L* < subtractions). A possible explanation for the higher delay in subtraction, revealed by regression, is that participants transiently represent the absolute value of the subtrahend. One additional processing stage may therefore be involved in subtractions: the conversion of the absolute value of the subtrahend into a negative number. Additionally, within addition problems, a possible explanation for the difference between *L* + *S* and *S* + *L* additions is that the selection of the smaller operand is faster when the numbers are in the appropriate left-to-right order.

Most importantly, we discovered a possible origin of the *Min* effect, reflected by a serial influence of the size of the second operand on finger trajectories. During an operation such as 9 - 8, the finger did not instantaneously point to the result 1, but slowly veered toward it, successively pointing to each intermediate location 9 - 0, 9 - 1, 9 - 2, and so on. Analysis confirmed that, when the second operand increased, the trajectories diverged at increasingly later times: 9 - 8 diverged from 9 - 7 at a later time than 9 - 7 diverged from 9 - 6, and so on. Thus, calculation always starts with the larger operand and then the correct result is attained in a slow and incremental manner, proportional to the *Min* operand, with a slope of about 50–60 ms per unit.

The two serial stages that we identified—first point to the larger operand, then serially deviate toward the target number—strongly constrain the models of mental arithmetic that we outlined in the introduction. Our results are most compatible with models where arithmetic operations are solved by serial quantity manipulation, possibly relying on a mental number line (Dehaene et al., 1999). They fit precisely with the original Groen and Parkman (1972) *Min* Model of addition, in which subjects start with the larger number and count up the smaller quantity. The slope of that serial process was thought to be too fast for counting, but the present results do suggest a slow and serial incremental process. Its fast speed suggests a series of “jumps” on the mental number line, where quantities are chained by successor and predecessor operations, rather than an explicit verbal counting process.

Our finger-tracking task could have biased subjects to use approximation or other quantity-based manipulations. This possibility is unlikely, however, given that our movement-time results converge with classical studies of the problem-size effect (Groen & Parkman, 1972) and two of its most recent reexaminations. Using a classical calculation task with oral responses, Barrouillet and Thevenot (2013) and Uittenhove et al. (2016) presented robust evidence that additions are solved using “fast procedures that scroll an ordered representation such as a number line.” The convergence of results suggest that the proposed model may not be restricted to the current experimental design, but may be generalized to other methods of measuring mental calculation. While previous studies could infer this underlying mechanism only indirectly, from the patterns of RT, our method provides a more direct look at the underlying processing stages.

The hypothesis of a mental displacement in quantity space also accounts for another aspect of the results, namely, the operational momentum (OM). As previously reported (McCrink et al., 2007), additions produced a transient bias toward larger numbers and subtractions toward smaller numbers. Our results reject the hypothesis that OM originates solely from post-calculation processes, when the retrieved result attracts attention left or right. Rather, the present work shows that the OM effect coincides with the processing of the second operand, suggesting that movement on the number line occurs during the single-digit calculation process. This result concurs with the only study that actually investigated the timing of the OM effect during calculation, using mouse tracking rather than finger tracking (Marghetis, Núñez, & Bergen, 2014). It also fits with other studies suggesting that the visual-spatial attention system is actively involved in arithmetic calculations (Knops, Thirion, et al., 2009; Knops, Viarouge, et al., 2009; Mathieu et al., 2016; McCrink et al., 2007).

Our results impose strong restrictions on retrieval-based models of single-digit additions (Ashcraft, 1992; Campbell, 1987; Siegler, 1987; Zbrodoff & Logan, 2005). These models postulate a direct access to a memory for arithmetic facts and therefore have no reason to postulate either a faster influence of the larger number, a linear effect of the smaller number, or an OM effect. Indeed, existing models of arithmetical retrieval typically assume that (1) the ease of retrieval depends on the frequency with which a problem has been encountered (Hamann & Ashcraft, 1986); (2) after a competition process, only a single result is ultimately selected (Ashcraft, 1992; Campbell, 1987; Siegler, 1987). Both properties fail to explain why the finger first points to the larger number, and then slowly veers toward the correct result, in proportion to the *min*.

In order to preserve the memory retrieval model, one would have to propose a table-search model (Ashcraft & Battaglia, 1978) according to which calculation time reflects a search for the proper entry in a stored table of arithmetic facts. For instance, for a problem like 6 + 3, subjects would first identify the larger number (6), then list all 6 + *x* problems, and finally search serially among them (6 + 1 = 7, 6 + 2 = 8, 6 + 3 = 9). Such a model is functionally equivalent to the above proposal, the only difference being that the movement occurs on a memorized table rather than a number line.

Overall, our findings highlight how a precisely timed series of operations underlies simple arithmetic. They also demonstrate that even complex mental operations can be continuously reflected in finger-pointing movements, as previously demonstrated in simpler cases (Song & Nakayama, 2009). Within the existing methods for investigating covert serial processes (King & Dehaene, 2014; Resulaj, Kiani, Wolpert, & Shadlen, 2009; Sternberg, 1969; Tanenhaus, Spivey-Knowlton, Eberhard, & Sedivy, 1995), finger tracking may play a special role as a simple and powerful behavioral method.

## ACKNOWLEDGMENTS

This research was sponsored by INSERM, CEA, and the Bettencourt-Schueller Foundation. DD is grateful to the Azrieli Foundation for the award of an Azrieli Fellowship. PPC gratefully acknowledges a Science Without Borders Fellowship from CNPq (no. 246750/2012-0).

## AUTHOR CONTRIBUTIONS

All authors conceived and designed the study. PPC collect the data. PPC analyzed the data with the support of DD. All authors helped interpret the data. PPC, SD, and DD wrote the manuscript.
