# Supplementary material for: Finger Tracking Reveals the Covert Stages of Mental Arithmetic
Source: Open Mind (Camb). 2017 Feb 1;1(1):30–41. doi: 10.1162/OPMI_a_00003 (PMC6436574; doi:10.1162/OPMI_a_00003)
Supplement: Supplementary file 1 [file opmi-01-30-s001.pdf]

Chernyak, N., Trieu, B. Y., & Kushnir, T. (2017). Preschoolers' selfish sharing is reduced by prior experience with proportional generosity. *Open Mind: Discoveries in Cognitive Science, 1*(1), 42–52. doi:10.1162/opmi_a_00004.

Supplementary Analyses

Full Regression Models

Experiment 1:

Response: Likelihood of Generous Sharing

	B	SE(*B*)	Wald	Odds Ratio	95% CI for Odds Ratio
**Condition***	**2.87**	**1.14**	**6.39**	**17.93**	**1.91 - 168.04**
Age	0.53	0.57	0.86	1.70	0.57 - 5.17
Gender (Female = 1)	0.21	0.66	0.10	1.23	0.34 - 4.47
Intercept	-4.42	2.58	2.94		

Experiment 2:

Response: Likelihood of Selfish Sharing

	B	SE(*B*)	Wald	Odds Ratio	95% CI for Odds Ratio
**Condition***	**-3.58**	**1.62**	**4.90**	**0.03**	**0.001-0.664**
Age	0.45	0.56	0.67	1.58	0.53 – 4.68
Gender (Female = 1)	-0.10	0.67	0.02	0.91	0.25 – 3.35
Intercept	-0.88	2.23	0.16		

Response: Likelihood of Fair Sharing

	B	SE(*B*)	Wald	Odds Ratio	95% CI for Odds Ratio
Condition	1.88	1.13	2.78	6.56	0.72 – 59.83
Age	-0.06	0.53	0.01	0.94	0.34 – 2.64
Gender (Female = 1)	-0.52	0.61	0.74	0.59	0.18 – 1.94
Intercept	-0.11	2.09	0.16		

Response: Likelihood of Generous Sharing

	B	SE(*B*)	Wald	Odds Ratio	95% CI for Odds Ratio
Condition	2.13	1.77	1.45	8.42	0.26 – 268.73
Age	-1.29	1.15	1.26	0.28	0.03 – 2.63
Gender (Female = 1)	1.88	1.21	2.42	6.52	0.61 – 69.22
Intercept	0.33	4.01	0.007		
